# Gene coexpression network analysis combined with metabonomics reveals the resistance responses to powdery mildew in Tibetan hulless barley

**DOI:** 10.1038/s41598-018-33113-7

**Published:** 2018-10-08

**Authors:** Hongjun Yuan, Xingquan Zeng, Qiaofeng Yang, Qijun Xu, Yulin Wang, Dunzhu Jabu, Zha Sang, Nyima Tashi

**Affiliations:** 1 State Key Laboratory of Hulless Barley and Yak Germplasm Resources and Genetic Improvement, Lhasa, 850002 China; 2 Research Institute of Agriculture, Tibet Academy of Agricultural and Animal Husbandry Sciences, Lhasa, 850002 China; 3 Wuhan Metware Biotechnology Co., Ltd, Wuhan, 430070 China; 4 Tibet Academy of Agricultural and Animal Husbandry Sciences, Lhasa, Tibet 850002 China

## Abstract

Powdery mildew is a fungal disease that represents a ubiquitous threat to crop plants. Transcriptomic and metabolomic analyses were used to identify molecular and physiological changes in Tibetan hulless barley in response to powdery mildew. There were 3418 genes and 405 metabolites differentially expressed between the complete resistance cultivar G7 and the sensitive cultivar Z13. Weighted gene coexpression network analysis was carried out, and the differentially expressed genes were enriched in five and four major network modules in G7 and Z13, respectively. Further analyses showed that phytohormones, photosynthesis, phenylpropanoid biosynthesis, and flavonoid biosynthesis pathways were altered during Qingke-*Blumeria graminis* (DC.) f.sp*. hordei* (*Bgh*) interaction. Comparative analyses showed a correspondence between gene expression and metabolite profiles, and the activated defenses resulted in changes of metabolites involved in plant defense response, such as phytohormones, lipids, flavone and flavonoids, phenolamides, and phenylpropanoids. This study enabled the identification of *Bgh* responsive genes and provided new insights into the dynamic physiological changes that occur in Qingke during response to powdery mildew. These findings greatly improve our understanding of the mechanisms of induced defense response in Qingke and will provide new clues for the development of resistant Tibetan hulless barley varieties.

## Introduction

Powdery mildew is a fungal disease that affects more than 10,000 plant species worldwide and significantly reduces the grain yields and quality of agricultural crops^1,2^. Powdery mildew caused by the obligate biotrophic ascomycete fungus *Blumeria graminis* (DC.) f.sp*. hordei* (*Bgh*) is a serious disease of wheat crops and can decrease grain yield by up to 30% depending on the severity of infestation.

Many plant species such as *Arabidopsis thaliana*^3^, wheat (*Triticum spp*.)^2,4^, rice (*Oryza sativa*)^5^, tomato (*Solanum lycopersicum*)^6^ and barley (*Hordeum vulgare*)^7^, have been frequently used for investigating the molecular mechanisms of plant resistance to fungal pathogens including the powdery mildews. It is now clear that plants mainly exploit a complex, two-tiered immune system to defend against pathogens^1,8,9^. Genome-wide transcriptome profiling has shown that a set of genes are significantly differentially expressed after attack by powdery mildew. In Arabidopsis, it has been estimated that approximately 14% of all annotated genes may be directly associated with pathogen defense^10^. In barley, a recent study identified 96 genes involved in resistance to non-adapted or adapted powdery mildew fungi^7^. Obviously, many genes participate in resistance signaling and defense processes against powdery mildew, and these genes may interact and be regulated in a complex manner.

Multi-omics techniques including genomics, transcriptomics, proteomics and metabolomics can be used to generate massive and complex data sets^11^. In combination, a systems biology approach has the potential to comprehensively investigate the complex biological processes that occur when plants respond to powdery mildew and define how these components dynamically interact. Therefore, a better understanding of the reconstruction of biochemical networks^12^ and complex signaling pathways can be obtained by the integration and analysis of omics data^13^.

Tibetan hulless barley (*Hordeum vulgare* L. var. *nudum*), also called “Qingke” in Chinese and “Ne” in Tibetan, with highland adaptation to extreme environmental conditions, is the principal food for Tibetans and an important crop in the Tibetan Plateau^14^. One of the major diseases of Tibetan hulless barley is powdery mildew caused by *Bgh*, and the cultivation of *Bgh*-resistant crop cultivars is the most economical method for controlling the damage. A previous study showed that Qingke varieties exhibited different responses to powdery mildew, suggesting that there is extensive genetic variation to explore^15^. Our limited understanding of Tibetan hulless barley genetics seriously hindered the systematical investigation of genes and molecular mechanisms underlying its resistance response to powdery mildew. Fortunately, using a whole-genome shotgun strategy, we built a 3.89-Gb draft genome of Tibetan hulless barley and predicted 36,151 protein-coding genes to better understand its adaptation to various stressful environments on highland and to facilitate crop improvement^14^. Furthermore, a set of genes involved in plant environmental responses and adaptation, such as plant hormone signal transduction and plant-pathogen interaction, were found to be positively selected in Qingke. However, the gene networks and molecular interactions involved in Tibetan hulless barley resistance to *Bgh* remain largely unknown. To efficiently breed resistant varieties, it is necessary to better understand how this plant responds to powdery mildew and then use this knowledge to improve the ability to cope with it.

An important object of gene expression studies is to reveal the underlying transcriptional networks, genes and pathways that are mediating a physiological process^16^. Gene coexpression network (GCN) analysis is a powerful systems biology approach whereby modules of highly coexpressed or connected genes can be identified and provide a meaningful way to examine the correlations in gene expression generated from complex RNA-seq datasets across developmental stages, treatments and time courses. A GCN is a set of relationships between genes where a node is defined as a gene connected to other genes by edges based on pairwise similarities^16^. A weighted gene coexpression network approach (WGCNA) assigns weights to the edges based on the strength of the correlation and can be used for finding modules of highly correlated genes, for summarizing clusters using the module eigengene or an intramodular hub gene^17^. WGCNA analysis of 10 Arabidopsis ecotypes following cold, heat, high-light, salt and flagellin treatment either separately or in combination identified several stress regulatory modules as important mediators of the regulatory response^18^. One of the important goals of network analysis is to connect gene expression data to the trait or stress response phenotype. Hub nodes have been found to play vital roles in many networks. Highly connected hub genes are expected to play an important role in biology but not always be significantly associated with the trait of interest^19^. It has been suggested that intramodular hub genes that are highly connected within a module are more likely to be biologically significant if that module is associated with the trait^19,20^.

Plants can biosynthesize specialized metabolites to adapt to various stresses such as biotic and abiotic stresses^21^. It has been suggested that indole metabolite, jasmonic acid (JA)^22^, salicylic acid (SA)^23^, callose^3^, phytoalexins, resveratrol and phenolic metabolites^24^, polyamine^25^, phenylpropanoids and flavonoids are all important compounds associated with plant responses to powdery mildew. To understand the underlying mechanism of crops’ stress responses and resistances, researchers have focused on the signaling perception, transcriptional regulation, functional protein expression and biosynthesis of specialized products in plants. Multi-omics analysis has become a powerful approach to identify gene-to-metabolite correlations^26^. The integration of modern omics technologies provides a great opportunity for better understanding plant defense mechanisms against powdery mildew at molecular and cellular levels. Previous studies have suggested that plant response to powdery mildew is a dynamic process, and that gene expression and metabolite accumulation are temporally regulated^7^. This information suggests that it is essential to investigate the dynamic changes at transcriptional, proteomic and metabolic levels during the plant response to powdery mildew. Transcriptomic studies can predict changes in gene expression, while metabolomic studies can investigate altered functions triggered by these genes or proteins. Therefore, the combination of transcriptomic and metabolic approaches is essential for a deeper understanding of plant responses to powdery mildew.

Here, to identify the changes that occur in the crop Tibetan hulless barley in responses to powdery mildew at molecular and physiological levels, we applied a transcriptomic coexpression network approach combined with metabolic analysis to associate gene expression with physiological responses. Coupled analysis of transcriptome and metabonomic parameters was performed over a long-time course (168 h) in a complete resistance cultivar Gannongda7 (G7) and a sensitive cultivar Zangqing13 (Z13). There were 3418 genes and 405 metabolites differently regulated during the plants’ interactions with *Bgh* between the two cultivars. WGCNA analysis showed that the differentially expressed genes were enriched in five and four major network modules in G7 and Z13, respectively. Further analyses showed that phytohormones, photosynthesis, phenylpropanoid biosynthesis and flavonoid biosynthesis pathways were altered during Qingke-powdery mildew interaction. Comparative analyses showed a correspondence between gene expression patterns and metabolite profiles, and the activated defenses resulted in changes of metabolites involved in plant defense response, including phytohormones, lipids, flavone and flavonoids, phenolamides, and phenylpropanoids. In addition, characterization of the transcription factors demonstrated that the WRKY transcription factor family exhibited a different regulation mechanism in the two cultivars and may play an important role in the response to powdery mildew. Gene coexpression network analysis combined with metabonomics in this study enabled the identification of powdery mildew responsive genes based on their differential expression profiles and provided new insights into the dynamic physiological changes that occur during the continuous process of Tibetan hulless barley response to powdery mildew.

## Results

### RNA sequencing and differential expression analysis

Powdery mildew can be observed as small grayish patches of fluffy fungal growth on the upper surface of the leaves. These spots resemble small cushions of white powder. The fungus only infects the epidermal layer of the leaf, and the tissue on the opposite side of an infected leaf turns pale green to yellow. Leaves remain green and active for some time following infection and then gradually become chlorotic and die off. G7 is a Qingke cultivar with complete resistance to powdery mildew, while Z13 is a sensitive cultivar. At the two-leaf and one needle stage, the well-developed seedlings were selected and inoculated with *Bgh*. Subsequently, leaves from each individual plant were collected at 0, 6, 36, 72 and 168 h post inoculation (hpi).

Subsequently, RNA-sequencing was conducted for the two Qingke cultivars G7 and Z13. RNA samples extracted from the leaves were collected at 0 (harvested prior to *Bgh* inoculation), 6, 36, 72, and 168 hpi, respectively. After adaptor sequence trimming and low-quality read filtering, a total of 2,035,104,484 high-quality reads were obtained for all Qingke samples, which have been deposited in the SRA database (accession number: SRR7177893, SRR7177910, SRR7177903, SRR7177904, SRR7177905, SRR7177906, SRR7177901, SRR7177902, SRR7177895, SRR7177884, SRR7177883, SRR7177886, SRR7177885, SRR7177888, SRR7177887, SRR7177890, SRR7177896, SRR7177897, SRR7177898, SRR7177899, SRR7177892, SRR7177891, SRR7177900, SRR7177881, SRR7177882, SRR7177889, SRR7177909, SRR7177908, SRR7177907, SRR7177894). For all 30 Qingke libraries, approximately 71.4% of the clean reads could be mapped to the Tibetan hulless barley reference genome sequence (http://show.genebang.com/project/download?n=barley) (Table S1).

**Figure 1:**
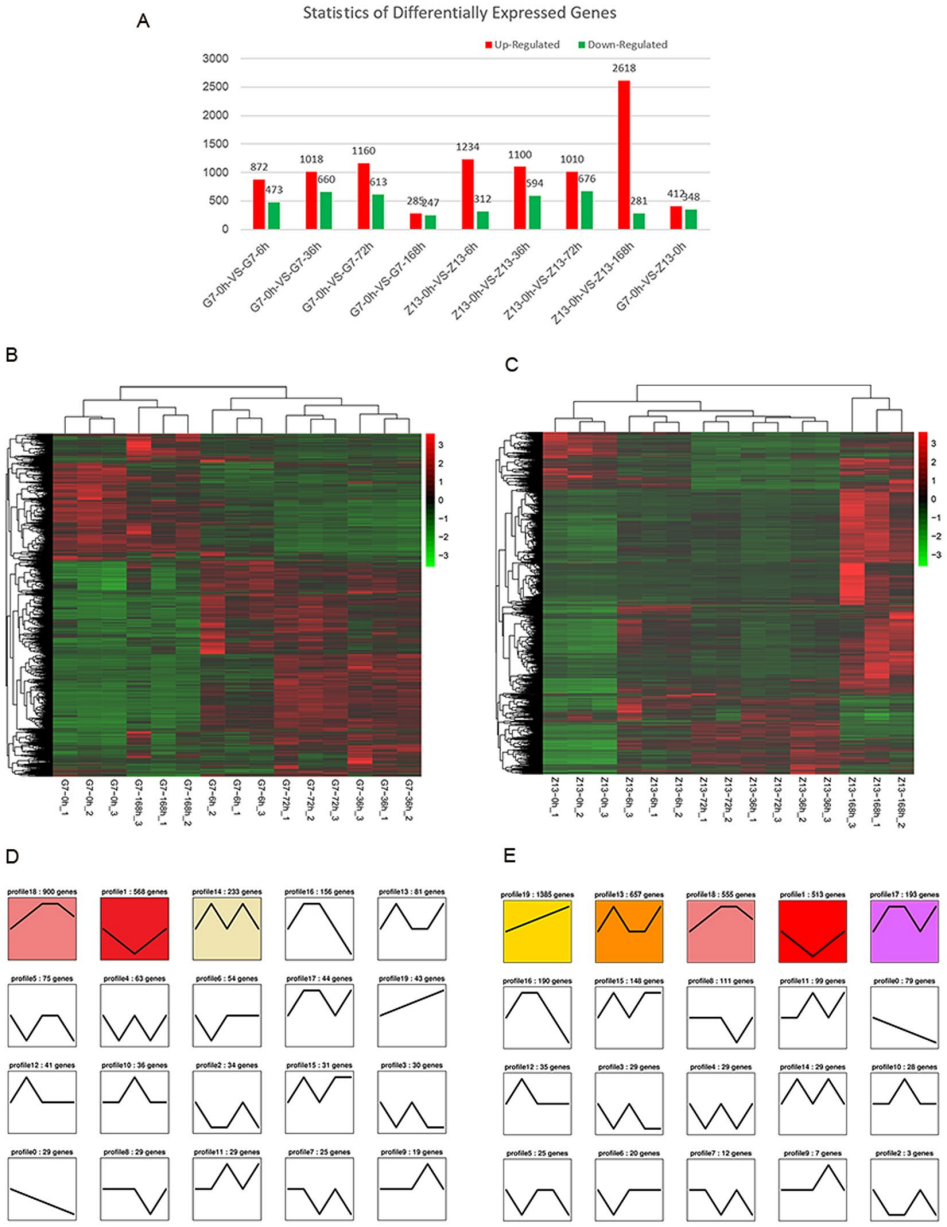
Differential gene expression of Tibetan hulless barley in response to powdery mildew. (**A**) Numbers of genes up-regulated (red) and down-regulated (green) in G7 and Z13 after inoculation with powdery mildew over time. (**B**) Heatmap of differentially expressed genes in G7. The columns show the 15 samples G7-0 hpi_1, G7-0 hpi_2, G7-0 hpi_3, G7-168 hpi_1, G7-168 hpi_2, G7-168 hpi_3, G7-6 hpi_1, G7-6 hpi_2, G7-6 hpi_3, G7-72 hpi_1, G7-72 hpi_2, G7-72 hpi_3, G7-36 hpi_1, G7-36 hpi_2, G7-36 hpi_3; while the rows show the TPM values scaled by the z-score algorithm. (**C**) Heatmap of differentially expressed genes in Z13. The columns show the 15 samples Z13-0 hpi_1, Z13-0 hpi_2, Z13-0 hpi_3, Z13-6 hpi_1, Z13-6 hpi_2, Z13-6 hpi_3, Z13-72 hpi_1, Z13-72 hpi_2, Z13-72 hpi_3, Z13-36 hpi_1, Z13-36 hpi_2, Z13-36 hpi_3, Z13-168 hpi_1, Z13-168 hpi_2, Z13-168 hpi_3; while the rows show the TPM values scaled by the z-score algorithm. Hierarchical clustering of expression pattern for genes in was shown at the left of the heatmap figure. (**D**) Clustering and classification of differentially expressed genes of G7 in response to *Bgh* over time (0, 6, 36, 72, 168 hpi). Twenty trends were determined, and the profiles of genes significantly enriched (P-value < 0.05) are colored. The number of genes in the trend is shown above each profile. (**E**) Clustering and classification of differentially expressed genes of Z13 in response to *Bgh* over time (0, 6, 36, 72, 168 hpi). Twenty trends were determined, and the profiles of genes significantly enriched (P-value < 0.05) are colored. The number of genes in the trend is shown above each profile.

In Qingke G7, 2520 genes were differentially expressed in the plant-pathogen interaction process (at 6, 36, 72, and 168 hpi) as compared with 0 hpi, and 165 genes were detected in all four time-points. Specifically, 872, 1018, 1160, and 285 genes were significantly upregulated and 473, 660, 613, and 247 genes significantly downregulated at 6, 36, 72, and 168 hpi, respectively (Fig. 1A).

In Qingke Z13, a total of 4147 genes were differentially expressed in the plant-pathogen interaction process (at 6, 36, 72, and 168 hpi) as compared with 0 hpi, and 411 genes were detected in all four time-points. In particular, 1234, 1100, 1010, and 2618 genes were significantly upregulated at 6, 36, 72 and 168 hpi, respectively; in contrast, 312, 594, 676, and 281 genes were significantly downregulated (Fig. 1A).

The overall number of differentially expressed genes in Qingke G7 increased at 6, 36, and 72 hpi over time but suddenly reduced at 168 hpi (Fig. 1A). The pattern of differential gene expression in Qingke Z13 was strikingly different with the G7 cultivar, especially at 6 and 168 hpi. Taking the time-point of 168 hpi for example, 2618 and 281 genes were significantly up or down-regulated in Z13, respectively, while only 285 and 247 genes were significantly up or down-regulated in G7.

Significantly different responses to *Bgh* in Qingke between G7 and Z13 cultivars were observed in the heatmap depicting hierarchical clustering of the gene expression data (Fig. 1B,C). In Qingke G7, the expression level of the majority of differentially expressed genes at 168 hpi was much similar with these genes at 0 hpi (Fig. 1B); while in Z13, the directionality of gene differential expression at 168 hpi differed strikingly with that at 0 hpi (Fig. 1C). The similarity between G7 and Z13 in response to *Bgh* can also be seen in the heatmap that a set of genes are expressed similarly at 36 hpi and 72 hpi.

A set of genes with similar expression patterns are often functionally correlated. The trends of all the differentially expressed genes in G7 and Z13 were analyzed respectively by Short Time-series Expression Miner software (STEM). A total of 20 trends were obtained from the expression data (Fig. 1D,E), and 3 trends were enriched in G7, and 5 trends were enriched in Z13. Interestingly, there were 900 genes enriched in G7 (Fig. 1D) and 555 genes enriched in Z13 (Fig. 1E) in profile18, while 568 genes enriched in G7 (Fig. 1D) and 513 genes enriched in Z13 (Fig. 1E) in profile1 (Fig. 1E). Surprisingly, 372 genes and 335 genes were further found in common in profile18 and profile1 in G7 and Z13, respectively. This result indicated that a great number of common genes (accounting for a large proportion) with similar expression patterns were involved in the process of Qingke-powdery mildew interaction in the two different Qingke varieties.

**Figure 2:**
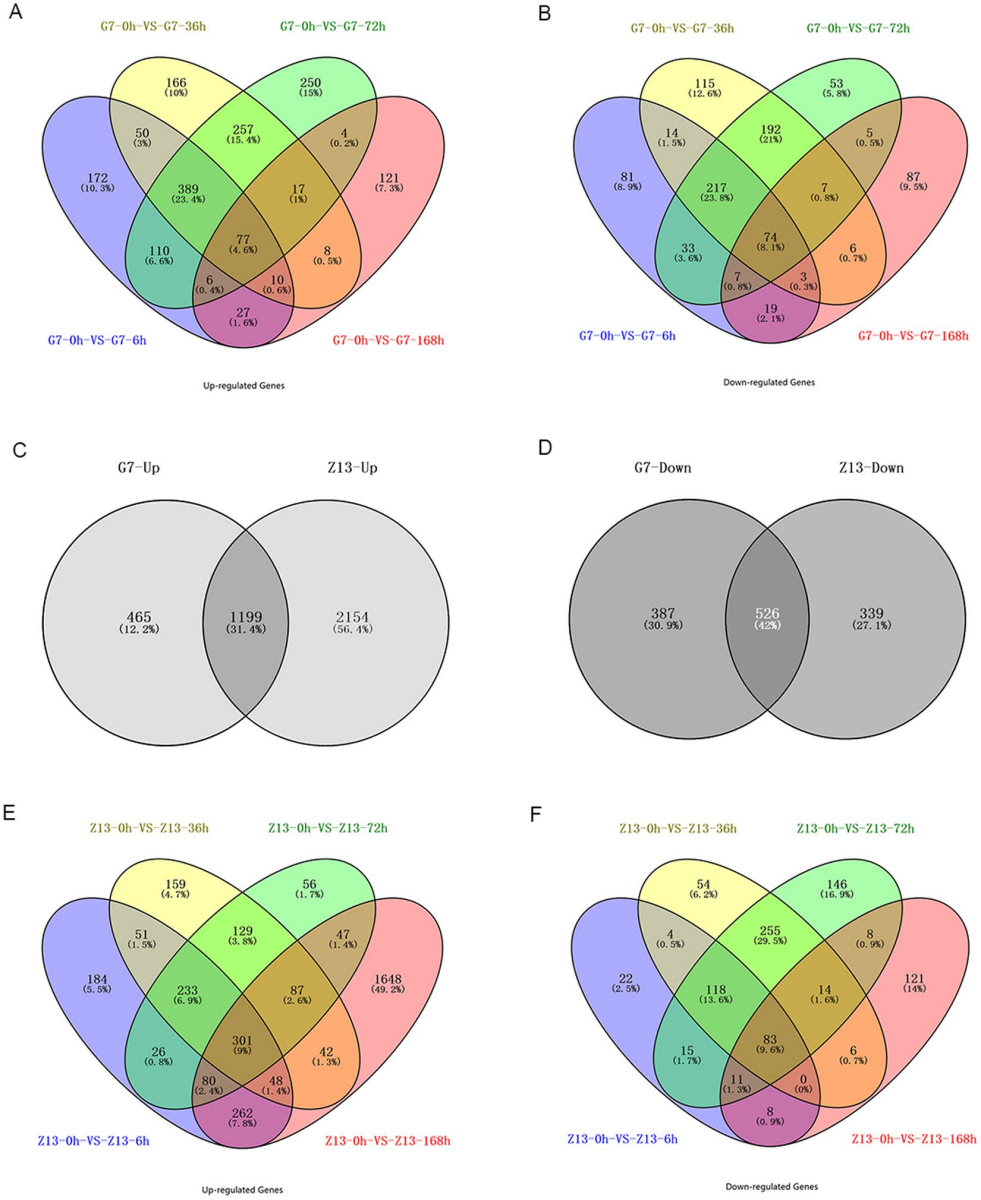
Venn diagram showing overlap of up-regulated and down-regulated genes of Tibetan hulless barley in response to powdery mildew. (**A**) Venn diagram showing overlap of up-regulated genes of G7 in response to powdery mildew. (**B**) Down-regulated genes of G7 in response to powdery mildew. (**C**) Venn diagram of the up-regulated genes of G7 and Z13 in response to powdery mildew. (**D**) Venn diagram of the down-regulated genes of G7 and Z13 in response to powdery mildew. (**E**) Venn diagram depicting intersections of up-regulated genes of Z13 in response to powdery mildew. (**F**) Down-regulated genes of Z13 in response to powdery mildew. Area of overlap is not proportional to the degree of overlap. The numbers of genes and their percentage in each region of the diagram are indicated.

In the G7 response to *Bgh*, 1664 genes were upregulated during at least one time-point and 913 were downregulated (Fig. 2A,B). Seventy-seven genes (4.6%) were upregulated at all time-points, while 466 genes (28.0%) were upregulated at 6, 36, and 72 hpi; 121 genes (7.3%) were upregulated only at 168 hpi (Fig. 2A). In addition, 74 genes (8.1%) were downregulated at all time-points, and 291 genes (31.9%) were downregulated at 6, 36, and 72 hpi (Fig. 2B). Interestingly, up regulated and down regulated genes were 1160 and 613 respectively at 72 hpi, while much fewer genes (285 up regulated and 247 down regulated genes) were identified at 168 hpi. This result might suggest that Qingke G7 had develop better adaptation and/or acquired stronger resistance to powdery mildew at 168 hpi.

In the Z13 response to *Bgh*, 3353 genes were upregulated during at least one time-point, and 865 were downregulated (Fig. 2E,F). Specifically, 301 genes (9%) were upregulated at all time points, while 534 genes (15.9.0%) were upregulated at 6, 36, and 72 hpi; surprisingly, 1648 genes (49.2%) were upregulated specifically at 168 hpi (Fig. 2E), which probably related to the severe disease development. Further, 83 genes (9.6%) were downregulated at all time points, and 201 genes (23.6%) were downregulated at 6, 36, and 72 hpi, while 121 were downregulated uniquely at 168 hpi (Fig. 2F).

Interestingly, 1199 (31.4%) up-regulated genes were in common between G7 and Z13 in their response to powdery mildew (Fig. 2C), and 526 (42%) down-regulated genes (account for 57.6% and 60.8% of the down-regulated genes in G7 and Z13, respectively) were in common between the two varieties (Fig. 2D). This result indicated that the two Qingke varieties triggered many of the same sets of genes and might have a similar resistant mechanism during the process of plant-powdery mildew interaction.

### Coexpression Network Analysis of Tibetan hulless barley in response to powdery mildew

**Figure 3:**
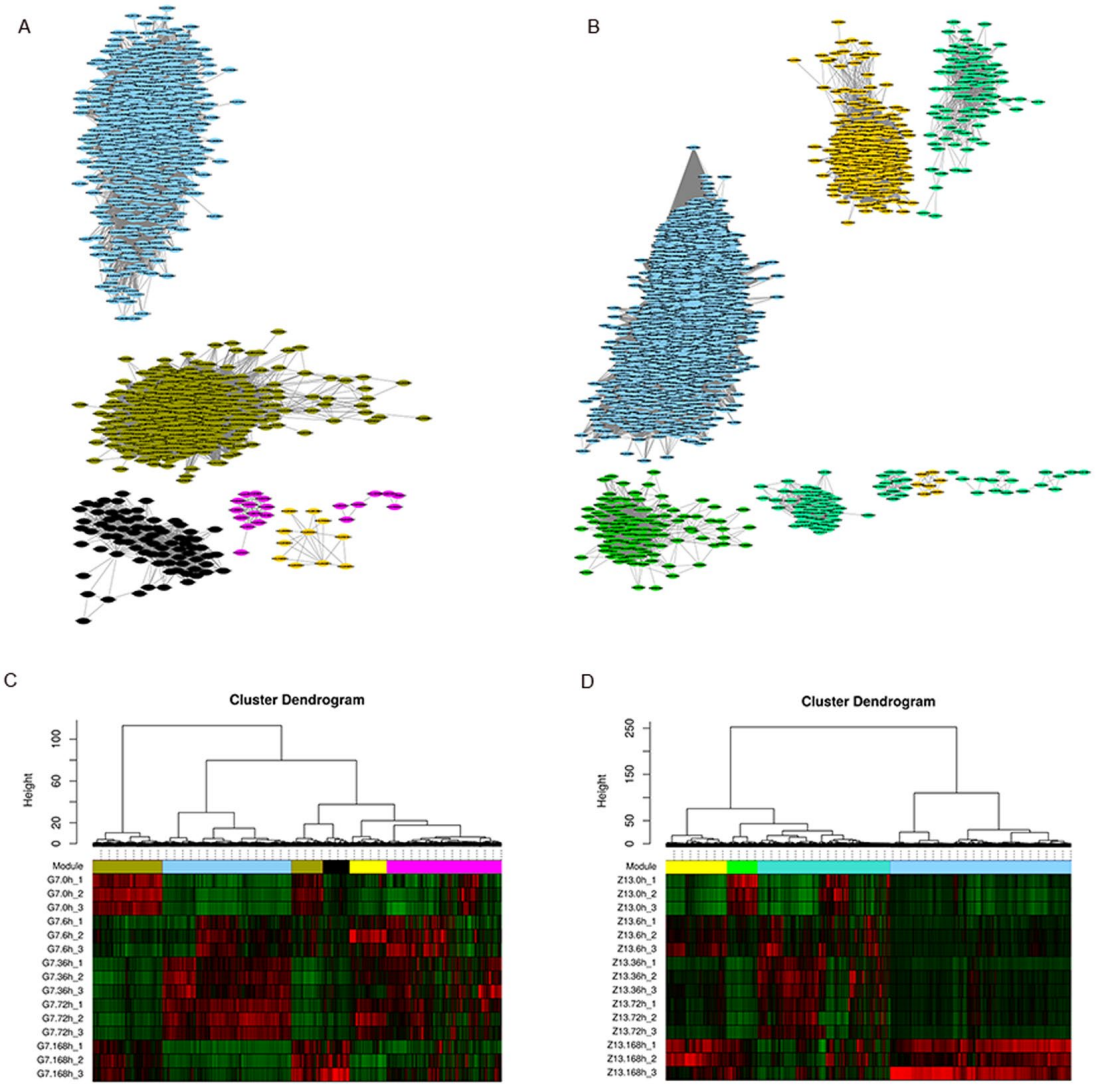
Weighted gene coexpression network of differentially expressed genes of G7 and Z13 in response to powdery mildew. (**A**) Weighted gene coexpression network of DEGs of G7 in response to powdery mildew. (**B**) Weighted gene coexpression network of DEGs of Z13 in response to powdery mildew. All adjacency values plotted are greater than 0.35. (**C**) Cluster of gene dendrogram in G7. Gene dendrogram and heatmap of the DEGs, showing 5 modules depicted with different colors. (**D**) Cluster of gene dendrogram in Z13. Gene dendrogram and heatmap of the DEGs, showing 4 modules depicted with different colors.

To further analyze the systematic transcriptional responses of Tibetan hulless barley to *Bgh* over time, we performed WGCNA based on data collected on the 2520 (G7) and 4147 (Z13) differentially expressed genes, respectively. GCNs are composed of genes that have similar profiles and are highly correlated with each other. The weighted interaction network is shown in Fig. 3A,B (Data S1). Nodes (genes) are connected by edges (coexpression relationships). The connection between two nodes was determined by the correlation between the expression levels of the genes those nodes represent across all experiments used in the analysis.

This analysis resulted in a network of G7 that grouped 2520 genes into 8 modules (Fig. S1), the most strongly interconnected of which are shown in Fig. 3A. Here, 5 unique modules were identified, with each module depicted with a different color. The module’s gene expression profile was represented by its eigengene (Fig. 3C), its most notable component. Notably, MEbrown comprised genes that were highly expressed (upregulated) at 0 hpi and 168 hpi, and MEblue included highly expressed genes at 36 and 72 hpi. MEblack were highly expressed uniquely at 168 hpi. For example, 164 genes involved in the MEblack module were highly expressed specifically at 168 hpi, which might indicate that this set of genes might be responsible for the process of resistance/adaption of G7 to powdery mildew. A total of 795 genes involved in the MEblue module were highly accumulated, specifically at 6, 36, and 72 hpi, which showed that this set of genes in the MEblue module might be responsible for the process of the early resistance/adaption of G7 to powdery mildew.

In Z13, a network that grouped 4147 genes into 4 modules (Fig. 3B) were identified, with each module depicted with a different color. The module’s gene expression profile was represented by its eigengene (Fig. 3D). Notably, MEgreen comprised genes that were highly expressed only at 0 hpi, with those of MEblue uniquely expressed at 168 hpi. Interestingly, a total of 1848 genes involved in the MEblue module were significantly upregulated specifically at 168 hpi, which might indicate that this group of genes might play important roles in the responses of Z13 to powdery mildew.

### Functional annotation

The integration of the functional annotations of genes comprising these modules with their expression profiles elucidate how the plant response to powdery mildew. AgriGO (http://bioinfo.cau.edu.cn/agriGO/analysis.php)^27^ was used to perform the function analysis and assign functions to the modules using the module gene lists. A collection of the GO-terms enriched in each module along with the relevant statistics is shown in Tables S2 and S3.

### Investigation of major modules correlated with Tibetan hulless barley response to powdery mildew

**Figure 4:**
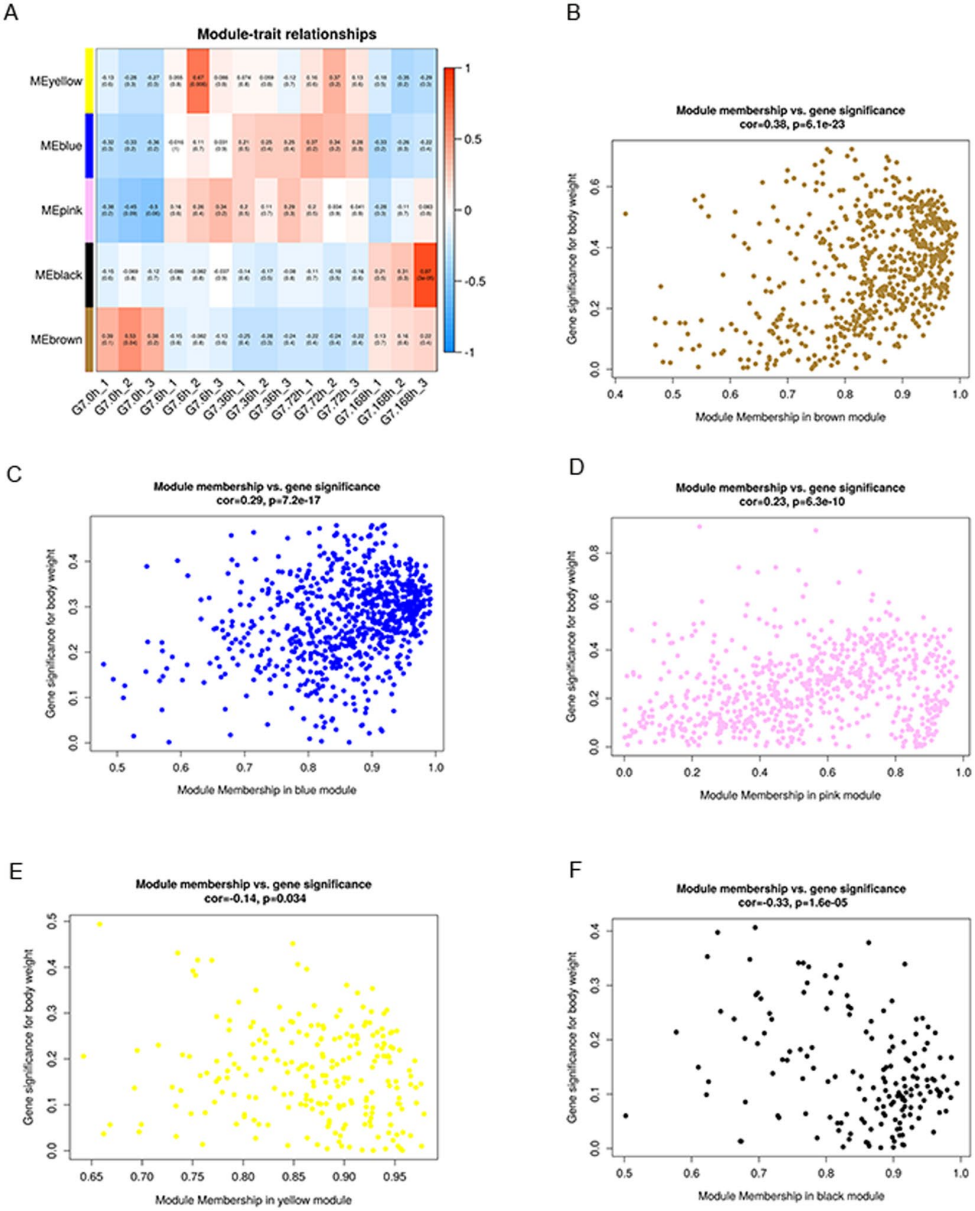
(**A**) Module-trait correlation of G7 in response to powdery mildew. Each row corresponds to a module. Each column corresponds to a specific time point. The color of each box at the row-column intersection indicates the correlation coefficient between the module and the time point. A high degree of correlation between a specific module and the time point is indicated by dark red or dark blue. (**B**–**F**) Scatterplots of gene significance versus each module membership.

As seen in the dendrogram on Fig. 3, each module’s gene expression profile is represented by its eigengene. In G7, the five resulting eigengenes correlate with the time points due to their gene expression profiles (Fig. 4A). Notably, 3 modules have similar expression patterns in the three time points (6 h, 36 h, and 72 h), including MEyellow, MEblue and MEpink. While MEblack was upregulated at 168 hpi, and MEbrown comprised genes that were upregulated at both 0 hpi and 168 hpi. Therefore, each of these modules identifies a set of genes of G7 in response to powdery mildew at specific time points.

For example, the MEbrown module had the highest correlation with G7, in which 350 genes were highly specifically expressed in G7 at 0 hpi and 168 hpi, which indicated that this set of genes might be responsible for the resistance of G7 to powdery mildew. KEGG pathway enrichment analysis showed that these genes were significantly enriched in phenylalanine metabolism, terpenoid backbone biosynthesis, sesquiterpenoid and triterpenoid biosynthesis, flavonoid biosynthesis, ubiquinone and other terpenoid-quinone biosynthesis, circadian rhythm-plant, zeatin biosynthesis, isoflavonoid biosynthesis and so on (Table S4).

**Figure 5:**
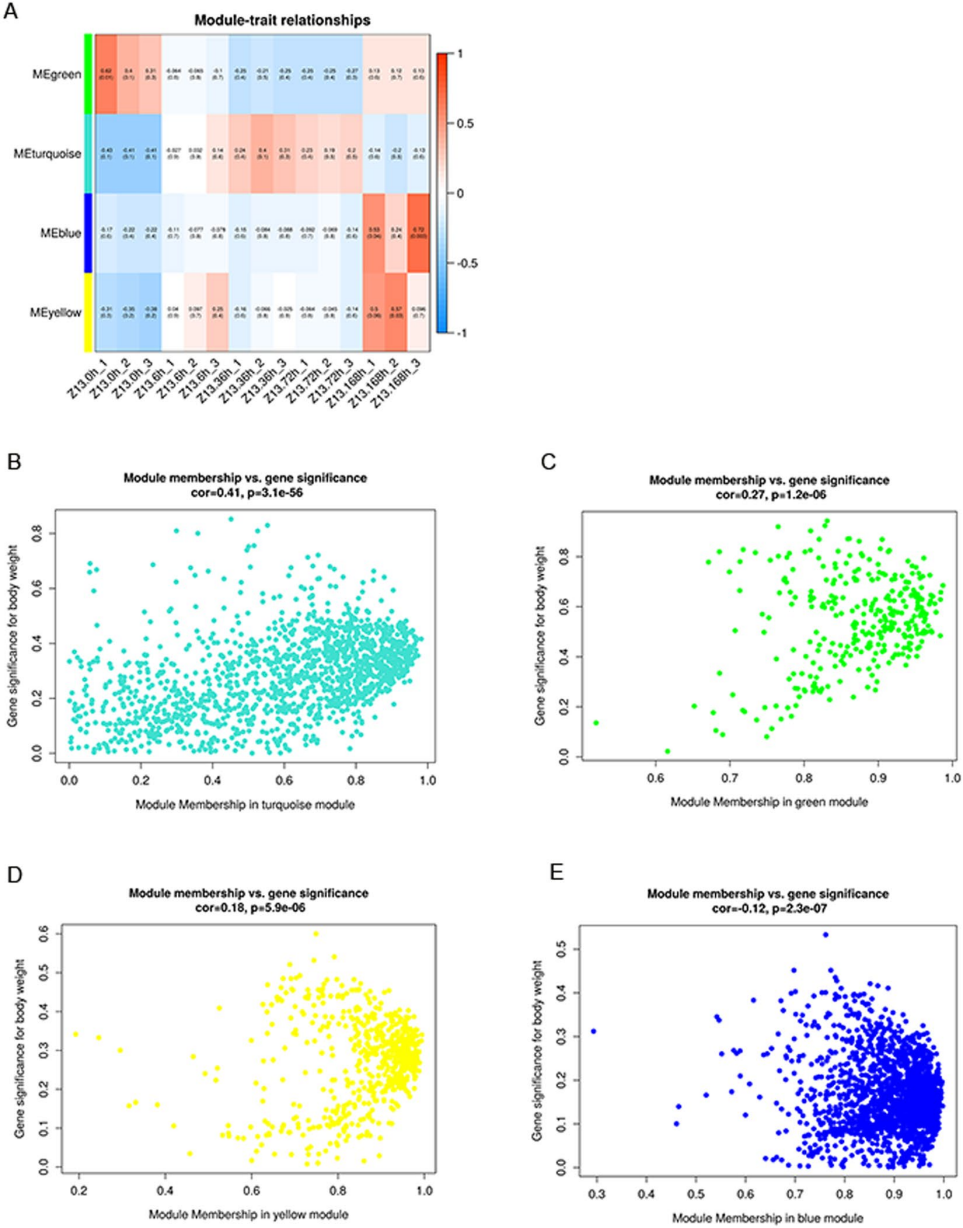
(**A**) Module-trait correlation of Z13 in response to powdery mildew. Each row corresponds to a module. Each column corresponds to a specific time point. The color of each box at the row-column intersection indicates the correlation coefficient between the module and the time point. A high degree of correlation between a specific module and the time point is indicated by dark red or dark blue. (**B**–**E**) Scatterplots of gene significance versus each module membership.

As seen in the dendrogram on Fig. 5A, each of these modules (MEturquoise, MEgreen, MEblue and MEyellow) identifies a set of genes in Z13 at specific time points. Notably, the MEturquoise module comprising genes that were upregulated in Z13 at 6, 36 and 72 hpi, had the highest correlation (cor = 0.41, *P = *3.1e-56) with Z13 in response to powdery mildew. MEblue and MEyellow comprised genes that were upregulated in Z13 at 168 hpi, while the MEgreen module comprised genes that were upregulated at the 0 hpi and 168 hpi.

As an example, 679 genes in the MEturquoise module were specifically enriched in photosynthesis, phenylpropanoid biosynthesis, circadian rhythm-plant, monoterpenoid biosynthesis, plant-pathogen interaction, plant hormone signal transduction, starch and sucrose metabolism, and flavonoid biosynthesis (Table S5), which indicated that this group of genes might be responsible for the responses of Z13 to powdery mildew. A set of genes involved in the MEblue and MEyellow modules were specifically upregulated at 168 hpi and enriched in proteasome, protein processing in endoplasmic reticulum, ABC transporters, pyruvate metabolism, citrate cycle (TCA cycle), glutathione metabolism and ribosome-related biological processes, indicating that these groups of genes might be involved in the later process of powdery mildew infection.

### Identification of differentially expressed Transcription factors (TFs)

In consideration of the important regulatory function of TFs in response to various stresses, we analyzed TFs-encoding genes by blast against the Plant Transcription Factor Database (PlnTFDB,V3.0) (http://plntfdb.bio.uni-potsdam.de/v3.0/)^28^. As a result, 275 differentially expressed TFs distributed in 39 families were identified (Table S6). These TFs mainly include the following families: MYB-related (24 genes), bZIP (basic region/leucine zipper motif) (23 genes), NAC (NAM, ATAF1-2, and CUC2) (22 genes), WRKY (18 genes), bHLH (basic helix-loop-helix) (17 genes), MYB (myeloblastosis) (17 genes), and Co-like (14 genes). More details of the identified differentially expressed TFs are provided in Table S6. The genes belonging to WRKY families were further analyzed because of their important roles in plant resistant response to pathogenic bacteria. As a result, 10 KRKY genes were differentially expressed in G7 and Z13, most of which were highly induced in G7 at 36 hpi, while in Z13 they were highly induced at 168 hpi. This result reveals the different transcriptomic regulation mechanism in Qingke varieties in response to powdery mildew.

### Widely targeted metabolome analysis

To further analyze the dynamic changes at the metabolite level of Tibetan hulless barley caused by gene expression regulation, widely targeted metabolome analysis was carried out. As a result, a total of 568 known and unknown metabolites were detected and quantified in Tibetan hulless barley during the interaction between plants and powdery mildew (Table S7). By mapping the Metware metabolite database (local database) and the general biochemical pathways based on KEGG, the metabolites were divided into several classes, including amino acids and their derivatives, lipids, nucleotides and their derivates, organic acid and its derivatives, flavone and flavonoids, phenolamides, phenylpropanoids, terpenoids, and phytohormones. (Table S7).

Principal component analysis (PCA) was further used to preliminary understand the overall metabolic differences between samples and variation between groups. The results show that there were differences among the Tibetan hulless barley samples at different time points and that there was an obvious variation tendency among the two Qingke groups, especially in Z13-0 hpi (Mcw01, Mcw02, Mcw03), Z13-168 hpi (Mcw13, Mcw14, Mcw15), G7-0 hpi (Mcw16, Mcw17, Mcw18), and G7-168 hpi (Mcw28, Mcw29, Mcw30) (Fig. S2). Interestingly, metabolic analyses showed that the levels of most amino acids, phenolamides, and lipids including glycerophospholipid and fatty acid were much higher in G7 than those in Z13 at the 0 hpi time-point. However, at the 168 hpi time-point, the levels of amino acids, amino acid derivatives, and phenolamides were much lower in G7 than those in Z13. The flavone and flavonoids were differentially accumulated at 0 hpi and 168 hpi in G7 and in Z13. The heatmap of metabolites detected in G7 and Z13 are shown in Fig. S3.

**Figure 6:**
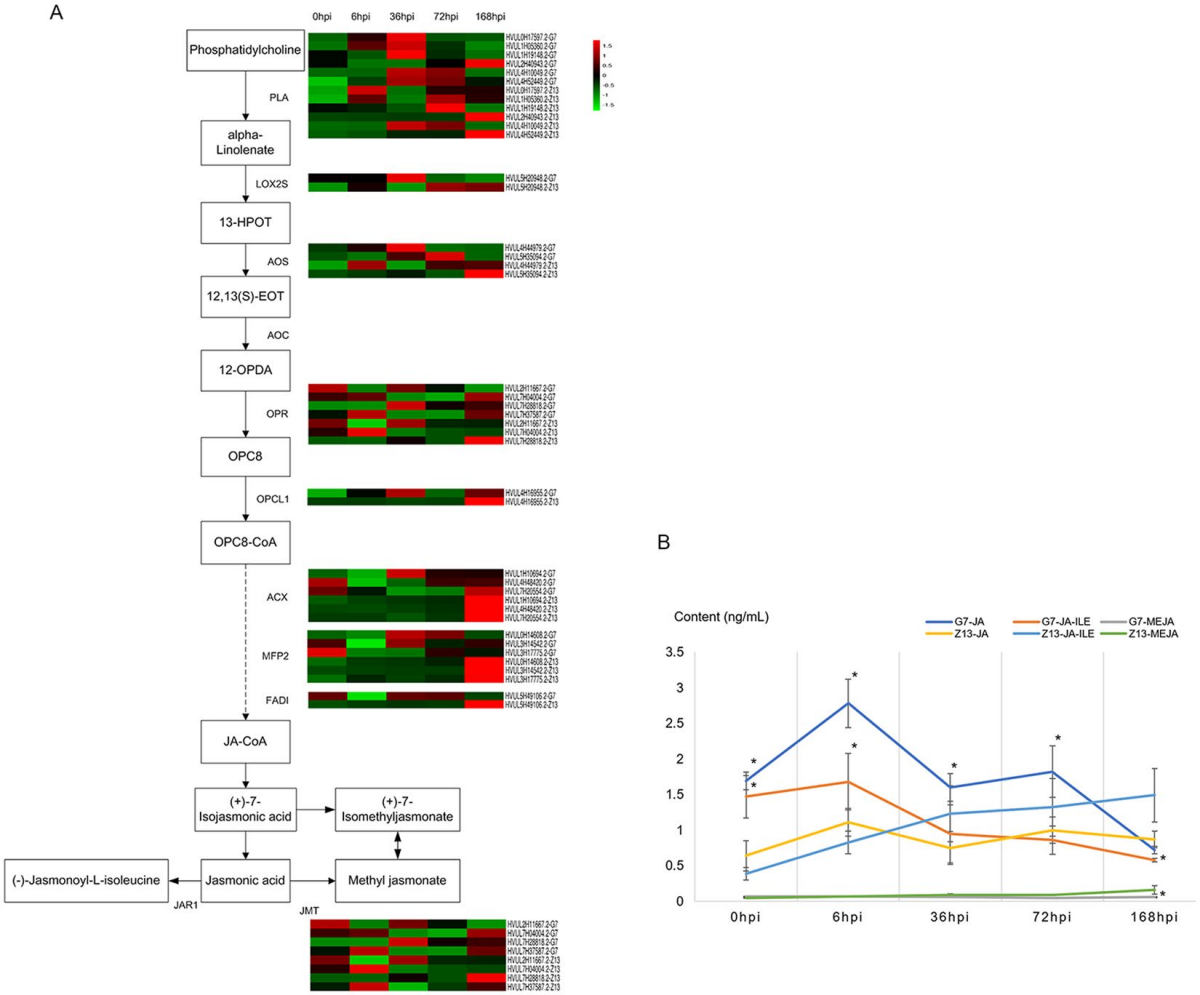
Expression patterns of genes and metabolites involved in the biosynthesis of jasmonates. (**A**) Schematic pathway. Uppercase letters indicate genes that encode enzymes. Solid arrows represent established biosynthesis steps, while broken arrows indicate the involvement of multiple enzymatic reactions. (**B**) Metabolite accumulation after *Bgh* inoculation; values are the means ± SE. The asterisk (*) indicates a significant difference in G7 versus Z13 (p < 0.05).

Jasmonates are critical components in mediating plant stress-induced systemic signals to activate defense-related genes. We found that differential expression of genes induced by *Bgh* in Qingke were correlated with changes at the physiological level. Our data showed that a set of JA-related genes were induced by *Bgh* at different time points, leading to the metabolite changes of jasmonates in G7 and Z13. (Fig. 6B).

At 0 hpi (un-inoculated control), the JA and JA-ILE levels were significantly higher in G7 (1.69 ng/mL and 1.47 ng/mL, respectively) than those in Z13 (0.64 ng/mL and 0.39 ng/mL, respectively), while MEJA was almost the same in the two cultivars. After inoculation with *Bgh*, endogenous JAs accumulated rapidly in G7 and reached the maxima at 6 hpi (2.78 ng/mL). Then, the JA content recovered to almost the same as that of the un-inoculated control at 36 hpi and 72 hpi and later decreased rapidly to 0.72 ng/mL at 168 hpi. However, in Z13, the concentration of JA accumulated to 1.11 ng/mL at 6 hpi and then changed slightly at 36, 72 and 168 hpi. Interestingly, the concentration of JA-ILE decreased gradually during the interaction in G7 with *Bgh*, from 1.47 ng/mL at 0 hpi to 0.58 ng/mL at 168 hpi, while in Z13, JA-ILE accumulated gradually from 0.39 ng/mL at 0 hpi to 1.49 ng/mL at 168 hpi.

### Integrated analyses of the transcriptomic and metabolic datasets

To gain a deep understanding of the resistant mechanism of G7 and Z13 based on their obvious resistance ability, we further analyzed the gene expression profile combined with the metabolomics analysis at the 0 hpi time point. We found that 412 and 348 genes were lower or higher expressed in Z13 compared with those in G7, and 71 and 59 compounds were differentially accumulated at the metabolic level. The enrichment analysis indicated that these DEGs were significantly enriched in glyoxylate and dicarboxylate metabolism, phenylpropanoid biosynthesis, monoterpenoid biosynthesis, alpha-linolenic acid metabolism, photosynthesis - antenna proteins, flavonoid biosynthesis, and starch and sucrose metabolism, among others (Table S8). The metabolite profiling showed that the differential metabolites mainly included phenylpropanoids and their derivatives, phenolamides, flavonoids, lipids, terpenoids and so on. Further correlation analysis of differential metabolites and DEGs at 0, 6, 36, 72, 168 hpi were carried out. The results showed that a great number of metabolites accumulated differentially during the process of Qingke-*Bgh* interaction, which were synchronous with the expression of the differentially expressed genes. 443 DEGs were identified to be related to the differential accumulation of 110 metabolites in the process of qingke response to powdery mildew (Table S9). This result indicated that the alteration in gene expression induced by powdery mildew was correlated with the metabolic changes at the physiological level during the process of Qingke-powdery mildew interaction.

## Discussion

To promote sustainability and productivity of plants in the face of powdery mildew, we must more efficiently develop resistant crops through gene modification, molecular breeding, or novel genetic-editing technologies. To effectively utilize these strategies to produce the next generation of crops, the multi-omics datasets should be appropriately exploited. The present study reports the first effort to integrate transcriptomic and metabolic techniques for the comparative analyses of the genes and the metabolites involved in responses of Qingke plants to powdery mildew. The results enhance our understanding of the mechanisms underlying the responses of Tibetan hulless barley to powdery mildew.

The knowledge of the plant-powdery mildew interaction ranges from well-defined pathways to a set of genes responsible for the resistance to *Bgh*^29^. A number of components of the plant-pathogen interaction pathway (ko04626) have been identified including pathogenesis-related protein 1 (PR1), LRR receptor-like serine/threonine-protein kinase (FLS2) (HVUL0H01543.2, HVUL0H00527.2, HVUL6H03320.2), mitogen-activated protein kinase kinase 1 (HVUL5H30208.2, HVUL5H06590.2), WRKY25/33 (HVUL7H41078.2, HVUL1H35549.2), disease resistance protein RPM1 (HVUL6H17646.2, HVUL7H06808.2, HVUL7H13517.2, HVUL1H07932.2, HVUL0H24172.2, HVUL6H27875.2, HVUL6H00968.2) and HSP90 (HVUL5H23218.2, HVUL7H37354.2). Interestingly, the overexpression of *CABPR1* in tobacco has recently been found to enhance resistance to pathogen stresses^30^. The characterization of 23 PR-1-like genes in hexaploid wheat suggested the diversity and conservation of *PR-1* gene functions in monocot plants, and revealed that 12 *TaPr-1* genes were induced or upregulated upon pathogen challenge^31^. In this study, the *PR1* genes (HVUL5H11228.2, HVUL0H09210.2, HVUL7H00882.2) were significantly induced (upregulated) in Z13, especially at the 168 hpi but were not differentially expressed in G7 at any time point, which might suggest that *PR1* plays an important role in Z13 when susceptible plants interact with powdery mildew.

Previous studies had shown that pathogens cause changes in gene expression that fluctuate over time. To investigate this further, WGCNA analyses were performed in the present study and revealed extensive temporal regulation of transcript levels in Tibetan hulless barley in response to powdery mildew.

Plant defenses to biotrophic pathogens include an inherent defense system and an induced system that is activated after plants detect the attack. The former mainly includes cutin, waxy and lignin of plant cell walls, and small molecule disease-resistant compounds (e.g. fatty acids, phenolic substances, terpenoids and flavonoids). In the WGCNA of G7, the MEbrown module has the highest correlation, in which 350 genes were significantly enriched in phenylalanine metabolism, terpenoid backbone biosynthesis, sesquiterpenoid and triterpenoid biosynthesis, flavonoid biosynthesis, zeatin biosynthesis, isoflavonoid biosynthesis and so on, indicating that this set of genes might be responsible for the resistance of G7 in response to powdery mildew. For example, 12 genes were identified in the phenylalanine metabolic pathway; most of these genes were highly expressed at 0 hpi and 168 hpi in both G7 and Z13, and these genes had similar expression patterns in both cultivars (Fig. S4). These results showed that the plants might trigger similar gene regulation mechanisms and response processes to cope with the infection of powdery mildew. Therefore, it can be proposed that plants employ the same set of genes in defense pathways during the process of this plant-pathogen interaction and that the sequentially differential expression of these genes contributes to the defense performance of the plant-pathogen interactions.

The plant cell wall is impermeable to water and pathogens and is dramatically affected by cutin, suberine and wax deposition^32^. The cutin-wax barrier plays a crucial role in the interaction and adaptation of plants to various stresses, particularly by providing the first barrier to many pathogens^33^. In the MEblue module of G7, four genes were identified to be involved in cutin, suberine and wax biosynthesis (HVUL0H23092.2; HVUL4H38244.2; HVUL4H27323.2; HVUL1H15328.2, Fig. S5) encoding fatty acid omega-hydroxylase, omega-hydroxypalmitate O-feruloyl transferase, peroxygenase and aldehyde decarbonylase, respectively. In G7, these genes were expressed at a low level at 0 hpi, were upregulated at 6, 36, and 72 hpi, and returned to the 0 hpi level at 168 h. However, in Z13, three genes were expressed at very low levels and were not differentially expressed throughout the process of interaction with *Bgh*. Interestingly, further metabolomics analysis showed that the primary substrates of cutin, suberine and wax biosynthesis, lipids including glycerophospholipids and fatty acids accumulated more in G7 at all time points. These results might suggest that these genes and metabolites play important roles in the complete resistance of G7 to powdery mildew.

Phytohormones play crucial roles in a complex regulatory network that is essential for pathogen-induced responses. Interestingly, our results showed that the content of jasmonic acid (JA) was much higher in G7 than that in Z13 at 0 hpi, and the expression of genes associated with JA biosynthesis was also further examined. The data showed that several JA related genes, such as OPR, ACX, MPF2 and FADI, were more highly expressed in G7 at 0 hpi than they were in Z13 (Fig. 6A). Many studies have reported that JA plays an important role in defense responses against biotrophic pathogens^34^; our findings are consistent with the conclusion that the accumulation of JA can have systematic effects on the resistance of powdery mildew in plants^22^.

Previous studies have suggested that the expression levels of *PR* are mediated through pathogen-induced signal-transduction pathways that are well regulated by phytohormones such as JA and MeJA^35^. The expression levels of 17 *PR*s were further analyzed in G7 and Z13 during the Qingke-*Bgh* interaction. Six genes (HVUL0H09210.2, HVUL3H06540.2, HVUL3H36018.2, HVUL5H11228.2, HVUL5H45926.2, HVUL7H00882.2) were induced by powdery mildew in G7 (Fig. S6), and the expression level of the *PRs* reached the maximum at 6 to 36 hpi. In the susceptive cultivar Z13, all 17 *PRs* were induced by powdery mildew (Fig. S6), and the expression level of the *PRs* reached the maximum at 168 hpi. The degree to which each *PR* gene was induced by *Bgh* differed in the two varieties. The result showed that the induced expression of *PR* genes was significantly different in G7 and Z13. The induced expression of *PR*s play more important roles in the resistant variety than in the susceptible one. These results indicated that powdery mildew infection had different effects on the 17 *PRs*, and the increased fold-changes in expression and the interval (response time) from inoculation to expression maxima were also different.

In conclusion, the combined transcriptome and metabolome analyses generated a set of data reflecting the dynamic defense responses of Tibetan hulless barley to powdery mildew. The defense responses involved primary metabolisms such as amino acid metabolism, carbohydrate metabolism, and lipids metabolism, and secondary metabolisms, including the biosynthesis of phenylpropanoids, flavone and flavonoids, phenolamides, and phytohormones. The gene expression and metabolic networks identified in this study provide new insights into the mechanisms of induced defense response in Qingke. The current findings will greatly improve our understanding of Qingke-*Bgh* interaction and provide clues for the development of resistant Tibetan hulless barley varieties.

## Methods

### Plant materials, *Bgh* inoculation and resistance

Two cultivated hulless barley G7 and Z13 were used in this study. G7 is a variety with complete *Bgh* resistance (showed complete penetration resistance to powdery mildew), while Z13 is sensitive to *Bgh*. In G7, after inoculated with *Bgh*, no significant changes in phenotype were observed during the long-time (168 h) process of Qingke-powdery mildew interaction. In Z13, the symptom of the disease is as follows: at 6 hpi, there was no significant changes; then at 36 hpi, there are tiny spots of fungal growth on the upper surface of the leaves. At 72 hpi, powdery mildew can be observed as little grayish spots and the spots become circular or irregular spots, which diameter reaches 1.0 mm. At 168 hpi, the patches connected into blocks and the spore density on the leaves were very thick with severe necrotic spots.

The hulless barley seedling cultivation and all experiments were carried out at the Tibet Academy of Agricultural and Animal Husbandry Sciences, Lhasa (Tibet, China). In the experiment, Qingke seedlings were grown in plant growth chambers under LD (18 h light:6 h dark) cycles (at 16–18 °C and a relative humidity of 80%). At the two-leaf and one needle stage, 30 well-developed seedlings were inoculated with *Bgh*. The growth temperature was adjusted to 20 °C after inoculation. The reactions to *Bgh* inoculation were observed visually based on the presence (or absence) of powdery mildew colonies and necrotic spots on the leaf surface. Subsequently, leaves from each individual plant were collected at 0, 6, 36, 72 and 168 hpi.

### RNA sequencing and preparation of high-quality transcriptomic reads

At each time point, Qingke leaf samples from five uniform seedlings were mixed for total RNA extraction using the Illumina TruSeq RNA Sample Prep Kit (Illumina, Inc., San Diego, USA). A total of 30 paired-end libraries were constructed and sequenced on Illumina HiSeq X Ten platform. High-quality reads were generated for each library. Genome mapping of high-quality reads was carried out using the software TopHat2. The Tibetan hulless barley draft genome assembly (http://show.genebang.com/project/download?n=barley) was used as a reference for genome mapping of the 30 Qingke libraries.

### Calculation of gene expression level and identification of differentially expressed genes

The expression levels of Tibetan hulless barley genes were measured using the TPM (transcripts per million) method, and low-expression genes with TPM <1 were filtered in all 30 samples by the Kallisto program^36^. The reference used was the coding sequence of 36,151 Tibetan hulless barley genes. Differentially expressed gene (DEG) analysis between samples was performed using NOISeq^37^. The probability of 0.8 and [log_2_ (Fold change)] greater than 1 were set as the thresholds for significant differential expression.

Gene expression pattern analysis was performed by Short Time-series Expression Miner software (STEM)^38^ on the OmicShare tools platform (www.omicshare.com/tools). The parameters were set as follows: (1) Maximum Unit Change in model profiles between time points is 1; (2) Maximum output profile number is 20 (similar profiles will be merged); and (3) Minimum ratio of fold change of DEGs is no less than two.

### WGCNA network analysis

The WGCNA R package was used to build GCNs, examine main network properties (hubs and modules), calculate the significance values of genes, and investigate the correlations between modules and powdery mildew resistance responses. The transcriptome datasets were filtered to remove any genes whose TMP value < 1 at any time point to remove these genes that introduce noise into the network analysis. Log_2_ normalized TMP values were used to construct the coexpression networks using the WGCNA package^17^ in R. Independent signed networks were generated from each Tibetan hulless barley cultivar G7 or Z13. The process of WGCNA network analysis was according to https://labs.genetics.ucla.edu/horvath/CoexpressionNetwork/Rpackages/WGCNA/.

### Widely Targeted Metabolome detection and data analysis

The freeze-dried leaf was ground using a mixer mill (MM 400, Retsch) with zirconia beads for 1.5 min at 30 Hz. 100 mg powder was extracted overnight at 4 °C with 1.0 ml 70% aqueous methanol. After centrifugation at 10, 000 g for 10 min, the extracts were absorbed and filtrated before LC-MS analysis.

The sample extracts were analyzed using an LC-ESI-MS/MS system (HPLC, Shim-pack UFLC SHIMADZU CBM30A system; MS, Applied Biosystems 4500 Q TRAP). The quantification of metabolites was accomplished using multiple reaction monitoring (MRM) analysis of a triple quadrupole-linear ion trap mass spectrometer (Q TRAP), API 4500 Q TRAP LC/MS/MS System. The analytical conditions and detailed operation parameters were as published before^39^.

For statistical analysis, the relative abundances of each metabolite were log transformed before analysis to meet normality. Dunnett’s test was used to compare the abundance of each metabolite between different time points. Statistical analyses were performed using the SPSS 22.0 software package (IBM SPSS, Somers, NY, USA). Differentially expressed metabolites were identified using a combination of fold change and the VIP values of the OPLS-DA model. The VIP values ≥1 and [log_2_ (Fold change)] greater than 1 were set as the threshold.

## Electronic supplementary material

Supplementary Information

Table S1

Table S2

Table S3

Table S4

Table S5

Table S6

Table S7

Table S8

Table S9

Dataset 1

## References

[1] Dodds PN, Rathjen JP (2010). Plant immunity: towards an integrated view of plant-pathogen interactions. Nature Reviews Genetics.

[2] Zhang J (2016). Coexpression network analysis of the genes regulated by two types of resistance responses to powdery mildew in wheat. Scientific Reports.

[3] Naumann M, Somerville S, Voigt C (2013). Differences in early callose deposition during adapted and non-adapted powdery mildew infection of resistant Arabidopsis lines. Plant signaling & behavior.

[4] Huang XQ, Röder MS (2004). Molecular mapping of powdery mildew resistance genes in wheat: A review. Euphytica.

[5] Cheng Y, Yao J, Zhang H, Huang L, Kang Z (2015). Cytological and molecular analysis of nonhost resistance in rice to wheat powdery mildew and leaf rust pathogens. Protoplasma.

[6] Seifi A (2013). Genetics and molecular mechanisms of resistance to powdery mildews in tomato (Solanum lycopersicum) and its wild relatives. European Journal of Plant Pathology.

[7] Douchkov D (2014). Discovery of genes affecting resistance of barley to adapted and non-adapted powdery mildew fungi. Genome biology.

[8] Chisholm ST, Coaker G, Day B, Staskawicz BJ (2006). Host-Microbe Interactions: Shaping the Evolution of the Plant Immune Response. Cell.

[9] Zipfel C (2009). Early molecular events in PAMP-triggered immunity. Current opinion in plant biology.

[10] Bevan M (1998). Analysis of 1.9 Mb of contiguous sequence from chromosome 4 of Arabidopsis thaliana. Nature.

[11] Bindschedler LV, Panstruga R, Spanu PD (2016). Mildew-Omics: How Global Analyses Aid the Understanding of Life and Evolution of Powdery Mildews. Frontiers in plant science.

[12] Droste P, Miebach S, Niedenführ S, Wiechert W, Nöh K (2011). Visualizing multi-omics data in metabolic networks with the software Omix: a case study. Bio Systems.

[13] Tieri, P., Fuente, A. D. L., Termanini, A. & Franceschi, C. *Integrating Omics Data for Signaling Pathways, Interactome Reconstruction, and Functional Analysis*. (Humana Press, 2011). 10.1007/978-1-61779-027-0_19 21370095

[14] Zeng X (2015). The draft genome of Tibetan hulless barley reveals adaptive patterns to the high stressful Tibetan Plateau. Proceedings of the National Academy of Sciences of the United States of America.

[15] Yuan, H. J. Identification of Powdery Mildew Resistance of Tibet Hulless Barley Germplasm. *Barley & Cereal Sciences* (2014).

[16] Gehan MA, Greenham K, Mockler TC, McClung CR (2015). Transcriptional networks-crops, clocks, and abiotic stress. Current opinion in plant biology.

[17] Steve H, Peter L (2008). WGCNA: an R package for weighted correlation network analysis. Bmc Bioinformatics.

[18] Rasmussen S (2013). Transcriptome Responses to Combinations of Stresses in Arabidopsis. Plant Physiology.

[19] Peter L, Mischel PS, Steve H (2013). When Is Hub Gene Selection Better than Standard Meta-Analysis?. PloS one.

[20] Horvath S, Dong J (2008). Geometric interpretation of gene coexpression network analysis. Plos Computational Biology.

[21] Nakabayashi R, Saito K (2015). Integrated metabolomics for abiotic stress responses in plants. Current opinion in plant biology.

[22] Chehab, E. W. & Braam, J. *Jasmonates in Plant Defense Responses*. (Springer Berlin Heidelberg, 2012).

[23] Kuhn H (2017). Key Components of Different Plant Defense Pathways Are Dispensable for Powdery Mildew Resistance of the Arabidopsis mlo2 mlo6 mlo12 Triple Mutant. Frontiers in plant science.

[24] Pociecha E, Janeczko Z, Janeczko A (2014). Resveratrol stimulates phenolic metabolism and PSII efficiency in wheat infected with powdery mildew. Journal of Plant Interactions.

[25] Cowley T, Walters DR (2010). Polyamine metabolism in barley reacting hypersensitively to the powdery mildew fungus Blumeria graminis f. sp. hordei. Plant Cell & Environment.

[26] Saito K (2013). Phytochemical genomics–a new trend. Current opinion in plant biology.

[27] Tian Tian, Liu Yue, Yan Hengyu, You Qi, Yi Xin, Du Zhou, Xu Wenying, Su Zhen (2017). agriGO v2.0: a GO analysis toolkit for the agricultural community, 2017 update. Nucleic Acids Research.

[28] Pérezrodríguez P (2010). PlnTFDB: updated content and new features of the plant transcription factor database. Nucleic Acids Research.

[29] Douchkov D (2011). Convergent evidence for a role of WIR1 proteins during the interaction of barley with the powdery mildew fungus Blumeria graminis. Journal of plant physiology.

[30] Sarowar S (2005). Overexpression of a pepper basic pathogenesis-related protein 1 gene in tobacco plants enhances resistance to heavy metal and pathogen stresses. Plant Cell Reports.

[31] Lu S, Friesen TL, Faris JD (2011). Molecular characterization and genomic mapping of the pathogenesis-related protein 1 (PR-1) gene family in hexaploid wheat (Triticum aestivum L.). Molecular Genetics & Genomics.

[32] Serra O, Figueras M, Franke R, Prat S, Molinas M (2010). Unraveling ferulate role in suberin and periderm biology by reverse genetics. Plant signaling & behavior.

[33] Beisson F, Li-Beisson Y, Pollard M (2012). Solving the puzzles of cutin and suberin polymer biosynthesis. Current opinion in plant biology.

[34] Vijayan P, Shockey J, Lévesque CA, Cook RJ, Browse J (1998). A role for jasmonate in pathogen defense of Arabidopsis. Proceedings of the National Academy of Sciences of the United States of America.

[35] Misra RC, Sandeep, Kamthan M, Kumar S, Ghosh S (2016). A thaumatin-like protein of Ocimum basilicum confers tolerance to fungal pathogen and abiotic stress in transgenic Arabidopsis. Scientific Reports.

[36] Bray NL, Pimentel H, Melsted P, Pachter L (2016). Near-optimal probabilistic RNA-seq quantification. Nature Biotechnology.

[37] Tarazona S, Garcíaalcalde F, Dopazo J, Ferrer A, Conesa A (2011). Differential expression in RNA-seq: a matter of depth. Genome Research.

[38] Ernst J, Barjoseph Z (2006). STEM: a tool for the analysis of short time series gene expression data. Bmc Bioinformatics.

[39] Khan, W. A. *et al*. Lipidomic study reveals the effect of morphological variation and other metabolite interactions on the lipid composition in various cultivars of Bok choy. *Biochemical & Biophysical Research Communications* (2018). 10.1016/j.bbrc.2018.04.112 29673595

