# Supplementary material for: Gene coexpression network analysis combined with metabonomics reveals the resistance responses to powdery mildew in Tibetan hulless barley
Source: Sci Rep. 2018 Oct 8;8:14928. doi: 10.1038/s41598-018-33113-7 (PMC6175840; doi:10.1038/s41598-018-33113-7)
Supplement: Supplementary file 1 — Supplementary Information [file 41598_2018_33113_MOESM1_ESM.docx]

**Article title:** **Gene coexpression network analysis combined with metabonomics reveals the resistance response****s to powdery mildew in Tibetan hulless barley**

**Authors: Hongjun Yuan^1,2^, Xingquan Zeng^1,2^, Qiaofeng Yang^3^, Qijun Xu^1,2^, Yulin Wang^1,2^，Dunzhu Jabu^1,2^, Zha Sang^1,2^,** **Nyima Tashi^2,4*^**

**Supplementary Figures**


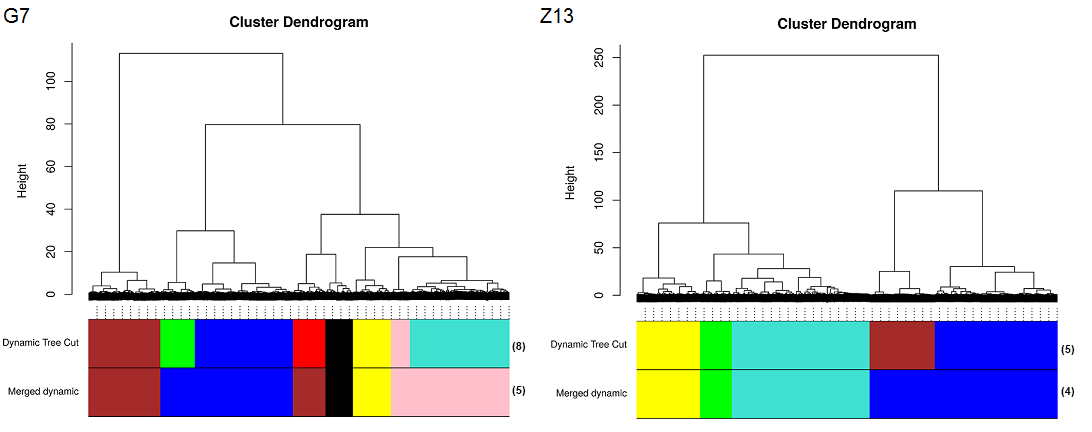


**Figure S1**. Hierarchical cluster tree showing co-expression modules identified by WGCNA analysis in G7 and Z13. The modules were represented by colorful bands. The number of modules in each GCN is given in the parentheses. The major tree branches constitute modules labeled by different colors.


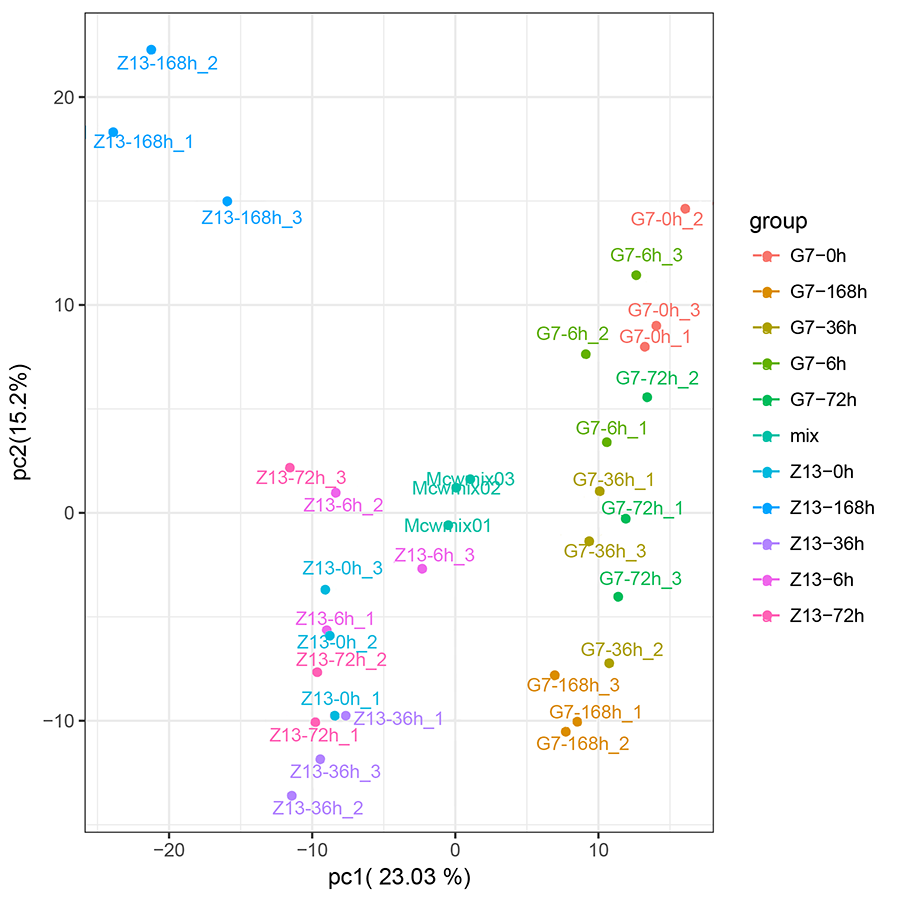


**Figure S2**. The PCA analysis of the metabolites in Qingke samples


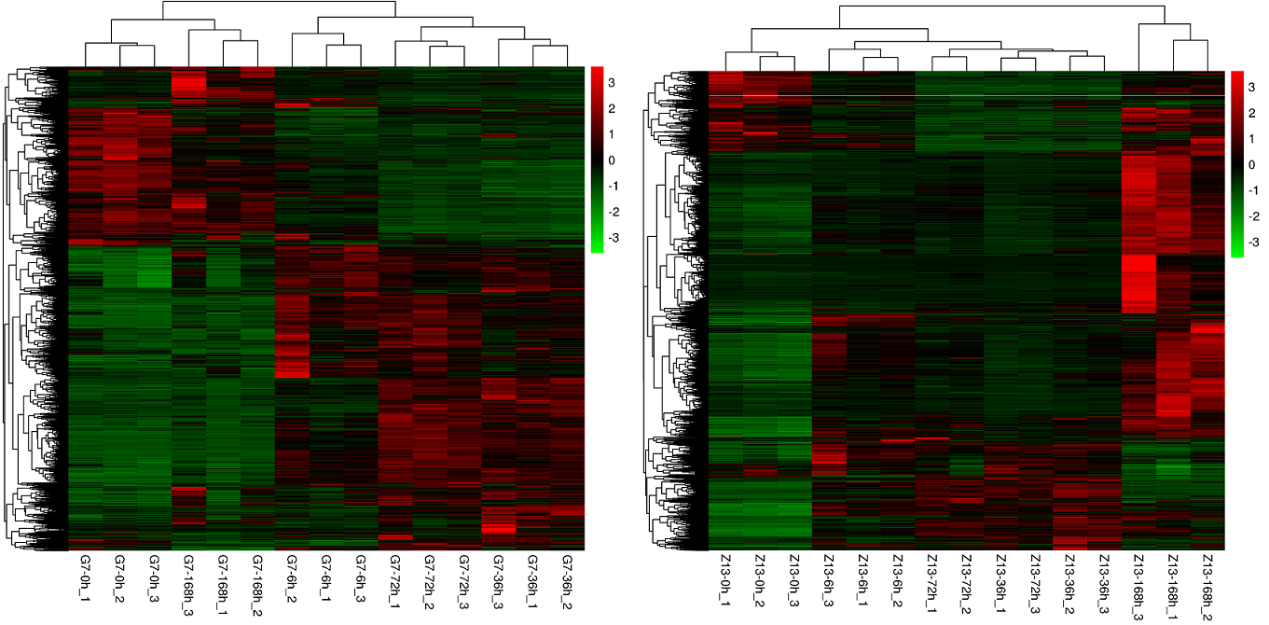


**Figure S3.** The heatmap of the metabolites levels in G7 and Z13. The columns show 15 samples of the two groups (G7 and Z13) at different time points; while the rows show the integral values of metabolites scaled by the z-score algorithm.


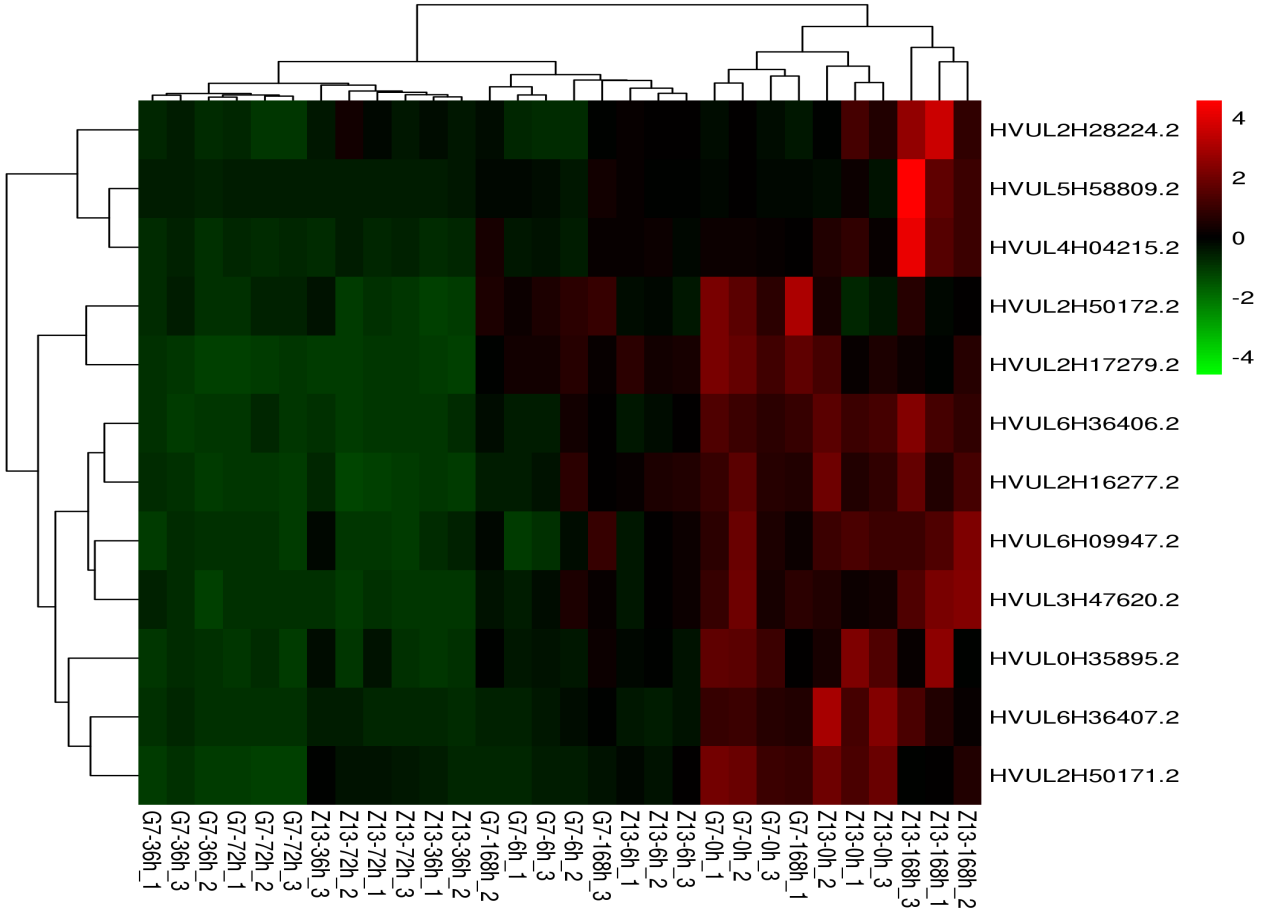


**Figure S4.** Heatmap of relative expression levels of the genes involved in phenylalanine metabolism. The columns show 30 samples of the two groups (G7 and Z13) at different time points; while the rows show the TPM values of each gene scaled by the z-score algorithm.


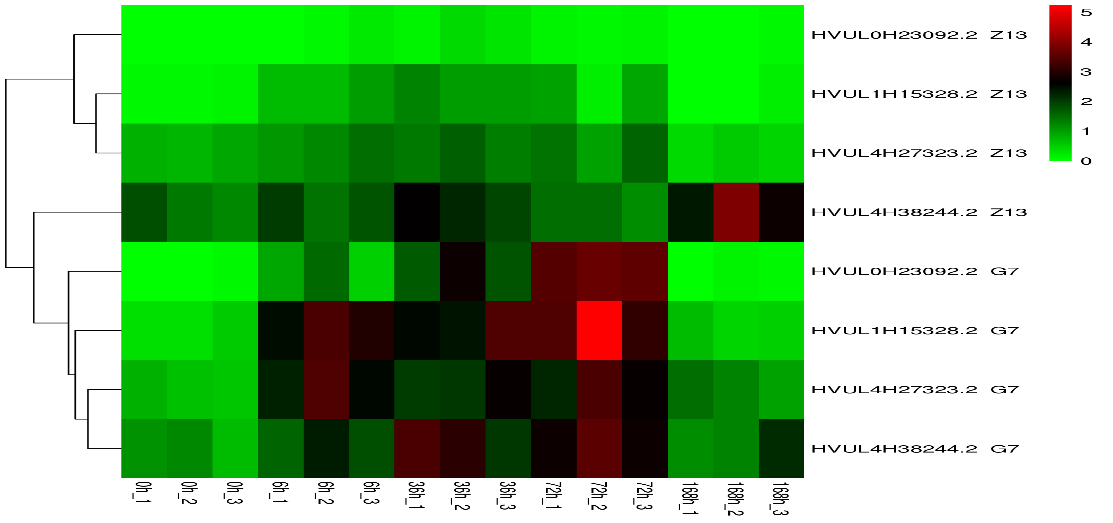


**Figure S5.** Heatmap of relative expression levels of the genes involved in cutin, suberine and wax biosynthesis. The columns show the different time points; while the rows show the TPM values of scaled by the z-score algorithm of the genes in the two groups (G7 and Z13).


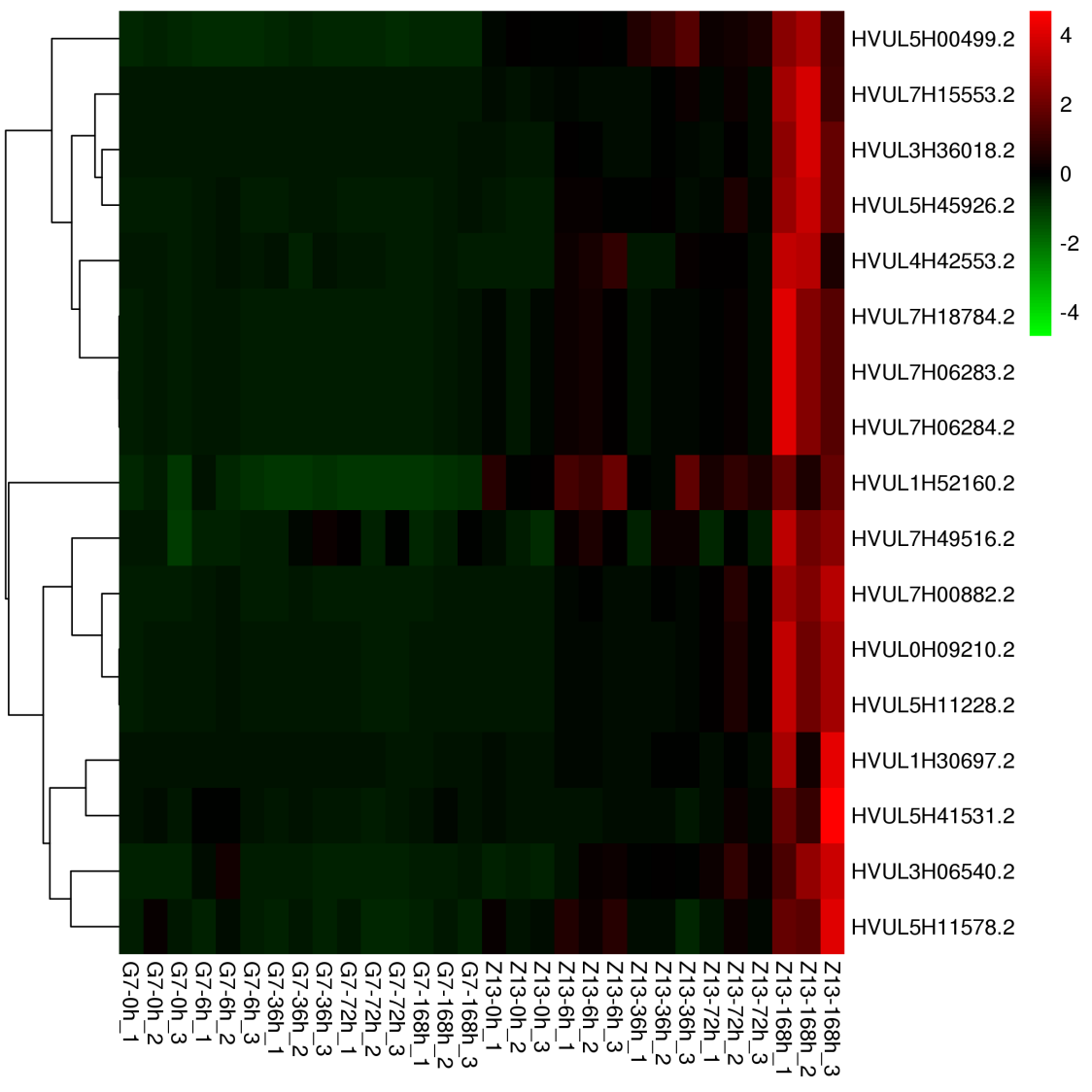


**Figure S6.** Heatmap of relative expression levels of the *PR* genes. The columns show 30 samples of the two groups (G7 and Z13) at different time points; while the rows show the TPM values of scaled by the z-score algorithm.

**Supplementary Tables**

Table S1. Summary of RNA sequencing and mapping using the Qingke genome.

Table S2. Specific GO terms enriched in the network modules of G7.

Table S3. Specific GO terms enriched in the network modules of Z13.

Table S4. KEGG pathway enrichment analysis of the genes in each module eigengene in G7.

Table S5. KEGG pathway enrichment analysis of the genes in each module eigengene in Z13.

Table S6. The differentially expressed Transcription factors.

Table S7. All metabolites detected by widely targeted metabolome analysis.

Table S8. List of significantly enriched pathways of the DEGs between Z13 and G7 at 0 hpi.

**Supplementary Data**

Data S1. The input files for the networks.
